# Improvement of surface ECG recording in adult zebrafish reveals that the value of this model exceeds our expectation

**DOI:** 10.1038/srep25073

**Published:** 2016-04-29

**Authors:** Chi Chi Liu, Li Li, Yun Wah Lam, Chung Wah Siu, Shuk Han Cheng

**Affiliations:** 1 Department of Biomedical Sciences, City University of Hong Kong, Hong Kong, China; 2 Department of Biology and Chemistry, City University of Hong Kong, Hong Kong, China; 3 Cardiology Division, Department of Medicine, Queen Mary Hospital, The University of Hong Kong, Hong Kong, China

## Abstract

The adult zebrafish has been used to model the electrocardiogram (ECG) for human cardiovascular studies. Nonetheless huge variations are observed among studies probably because of the lack of a reliable and reproducible recording method. In our study, an adult zebrafish surface ECG recording technique was improved using a multi-electrode method and by pre-opening the pericardial sac. A convenient ECG data analysis method without wavelet transform was also established. Intraperitoneal injection of KCl in zebrafish induced an arrhythmia similar to that of humans, and the arrhythmia was partially rescued by calcium gluconate. Amputation and cryoinjury of the zebrafish heart induced ST segment depression and affected QRS duration after injury. Only cryoinjury decelerated the heart rate. Different changes were also observed in the QT interval during heart regeneration in these two injury models. We also characterized the electrocardiophysiology of breakdance zebrafish mutant with a prolonged QT interval, that has not been well described in previous studies. Our study provided a reliable and reproducible means to record zebrafish ECG and analyse data. The detailed characterization of the cardiac electrophysiology of zebrafish and its mutant revealed that the potential of the zebrafish in modeling the human cardiovascular system exceeds expectations.

The zebrafish (*Danio rerio*) heart is a tubular structure with an atrium and a ventricle that is anatomically different to the human heart. Nonetheless the electrophysiology is remarkably similar. Unlike the relatively fast heartbeat of other animal models, the adult zebrafish heartbeat is 110 and 130 beats per min (bpm)^1^, and markedly closer to that of humans. In addition, the electrocardiogram (ECG) waveform presents a distinct P wave, QRS complex, and T wave^2^. The value of the QT interval is comparable with that in humans^2^, suggesting that depolarization and repolarization are also similar. ECG is a common diagnostic tool for human cardiovascular disease. The use of the zebrafish as an animal model that can reproduce and mimic the human ECG trace has been considered to provide a promising research tool for human cardiovascular disease research and in the pharmaceutical industry^3^,^4^.

Surface ECG recordings of adult zebrafish have been reported since 2006^2^. Similar to precordial leads (chest leads) in human ECG recording, a needle electrode is placed on the chest of the zebrafish, and the surface electrical signal is amplified and recorded. An *in vitro* recording of the explanted heart and an implanted microelectrode array for direct contact with the epicardium have also been reported^5^,^6^. Human ECG recordings can be affected by different noise sources, such as power line artifact, electrode contact noise, and muscle movement artifacts. In previous studies, the adult zebrafish ECG signal showed high variability with multi-morphology waveform and variation in QT and QTc intervals because of such interference (for review, see Table 1). The QT interval often varies between leads on the standard human ECG and the lead II recording is mainly used in QT interval measurement. Similar to experience in humans, the different placement of the electrode in adult zebrafish ECGs is thought to account for the variation in T wave morphology and amplitude between different fish^2^,^7^. Measurement of ECG intervals is of considerable importance because it provides an indirect measure of the state of the heart and can be indicative of the presence of certain cardiac conditions. QT interval is the most important timing interval in the ECG waveform, defined as the time from the upstroke of the QRS complex to the end of the T wave. This interval represents the duration of electrical activity (both depolarization and repolarization) of the ventricle in a given heartbeat. Nonetheless there is large inter-lab variation in adult zebrafish ECG measurement and the zebrafish ECG remains incompletely characterized. The reported QT intervals of adult zebrafish in previous studies ranged from 250 ms to 600 ms with multiform ECG signals (Table 1). Clearly a reliable and reproducible method to record and analyse the adult zebrafish surface ECG is critically needed.

In this study, the adult zebrafish surface ECG recording method was improved by using two recording needle electrodes. The effects of the dermis and pericardial sac in ECG recording are also discussed. The proposed method allows us to record a zebrafish ECG in the presence of hyperkalemia, and is remarkably similar to a human ECG during hyperkalemia. This finding indicates the better than expected potential of zebrafish as a model of human cardiovascular disease. We also investigated the change in ventricular repolarization during heart regeneration in an amputation and cryoinjury model and a zebrafish mutant as a human long QT syndrome model.

## Results

### Adult zebrafish surface ECG recording

**Figure 1:**
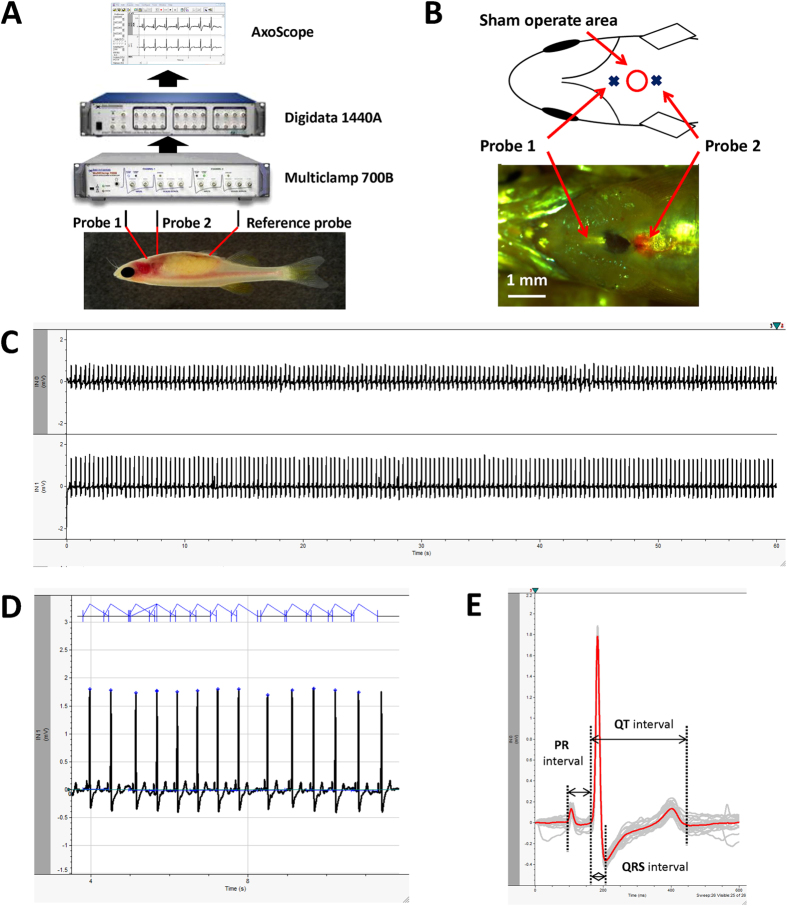
Component of Dual probe ECG recording system and data analysis method. (**A**) Scheme of ECG recording system. (**B**) Position of two recording probes. (**C**) Raw signal of 1 min gap free recording of adult zebrafish surface ECG. (**D**) Automatic identification and extraction of each heart beat cycle. (**E**) Overlay of extracted trace (Trace in gray) and measurement of ECG intervals based on average trace (Trace in red).

A noise-free raw signal with an isoelectronic baseline was obtained using our modified method (Fig. 1C–E). The zebrafish raw ECG signal was highly similar to a human ECG. The signal includes characteristic peaks, such as a P wave, QRS complex, and T wave, that can be easily identified without any signal processing, such as wavelet transform (Fig. 1D). Each heart beat cycle was identified and extracted from 1 min gap-free signal (Fig. 1D). Averaging of all extracted heart beat cycles (number or cycle depends on heart rate) further eliminated noise (Fig. 1E). Accurate ECG parameter can be measured on this trace. The mean heart rate of wild-type zebrafish was 118 bpm ± 14 bpm (*n* = 10). The mean PR and QRS intervals were 63.5 ± 7.2 and 35.0 ms ± 3.3 ms (*n* = 10), respectively. The mean QT interval was 282 ms ± 29 ms, and mean HR corrected QT interval (QTc) was 389 ms ± 38 ms.

### Dermis and pericardial sac effect on zebrafish ECG recording

**Figure 2:**
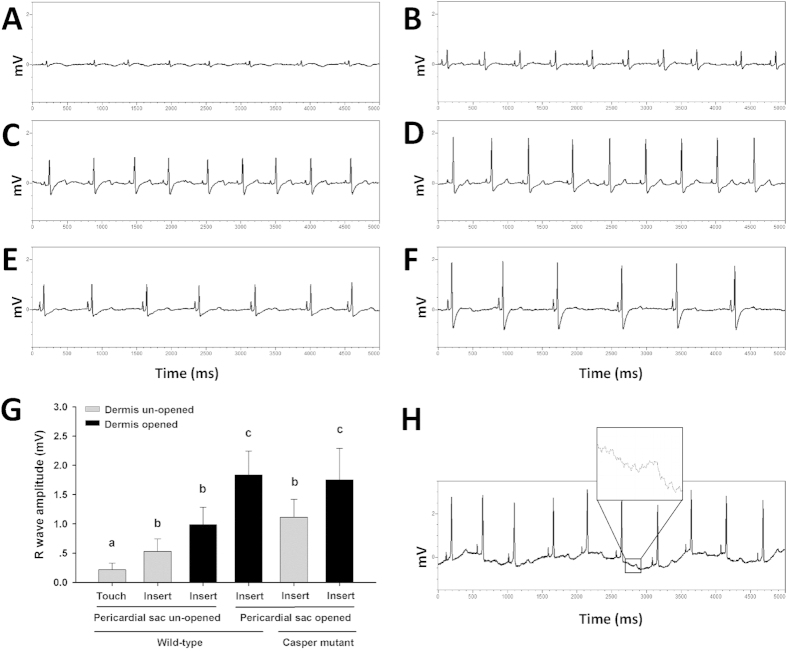
Effects of dermis and pericardial sac on adult zebrafish surface ECG measurement. Raw ECG signal of: (**A**) probes touching chest dermis, (**B**) probes inserted through the chest dermis, (**C**) Probes inserted into chest muscle layer without dermis. (**D**) Probes inserted into chest muscle layer after opening of pericardial sac and removal of dermis. (**E**) Probes inserted through the chest dermis of casper mutant zebrafish. (**F**) Probes inserted into chest muscle layer without dermis in casper mutant. (**G**) R wave amplitude of Probe 2 under conditions described above, n = 6 for each group, Values are presented as the mean + SD. Different letters indicate means that are significantly different (Tukey’s test, p < 0.05). (**H**) Raw ECG signal obtained by using 1 Hz–100 Hz band pass filter.

Although the scales covering the chest were removed prior to ECG recording in adult zebrafish, and the electrodes only touched the dermis, the raw ECG signal showed an extremely low amplitude R wave and high amplitude artifact (Fig. 2A). The amplitude of the R wave was unquantifiable because of baseline fluctuations (Fig. 2A). Once the electrodes were inserted through the dermis, the ECG baseline became isoelectric, and the R wave became distinct (Fig. 2B). The amplitude of the R wave significantly increased after the dermis over the chest area was peeled back (Fig. 2C,G). The T wave was easier to identify after the dermis was peeled back (Fig. 2B,C). The ECG signal recording from the muscle layer in group 4 became more distinct after the pericardial sac was opened. The T wave of group 4 became more clear (Fig. 2D), and the amplitude of the R wave signal was significantly increased in comparison with the other groups of wild-type zebrafish (Fig. 2G). When the dermis was un-opened, the signal of the surface ECG of casper mutant was higher than that of the pericardial sac un-opened wild-type zebrafish, although the difference was not significant on Tukey’s test (Fig. 2G). After the dermis was peeled, the signal of the surface ECG of casper mutant was similar to that of Group 4 where the pericardial sac had already been opened.

### Similarity of ECG recorded in the presence of hyperkalemia in adult zebrafish and humans

**Figure 3:**
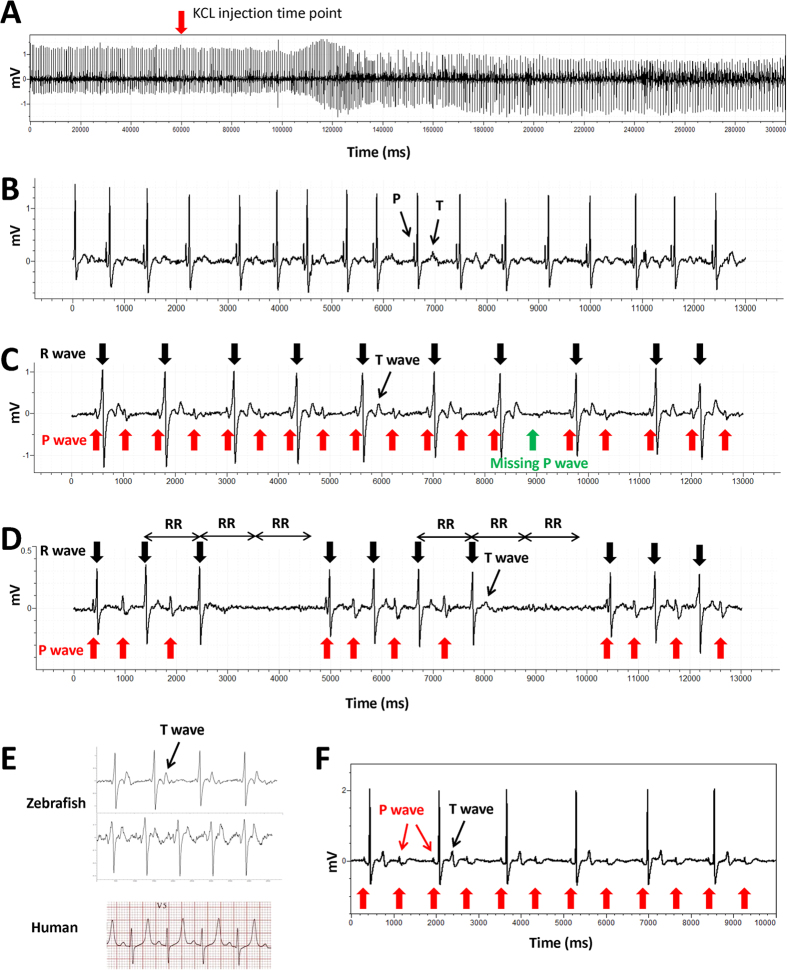
Hyperkalemia causes arrhythmia in adult zebrafish. (**A**) Simultaneous recording of ECG during intraperitoneal injection of KCL solution. (**B**) Raw signal showing normal ECG prior to injection of KCL solution. (**C**) Second degree AV block with 2:1 fixed ratio rhythm observed within 4 min post-injection. (**D**) AV block similar to human second degree AV block (Mobitz I) observed within 4 min post-injection. (**E**) Examples of zebrafish hyperkalemia ECG with widened QRS complex and peaked T wave (upper two) and human hyperkalemia ECG (lower) (adapted from http://lifeinthefastlane.com/ecg-library/basics/hyperkalaemia/). (**F**) Second degree AV block with 2:1 fixed ratio observed in all zebrafish specimens (*n* = 6) at 24 h post KCL injection.

**Figure 4:**
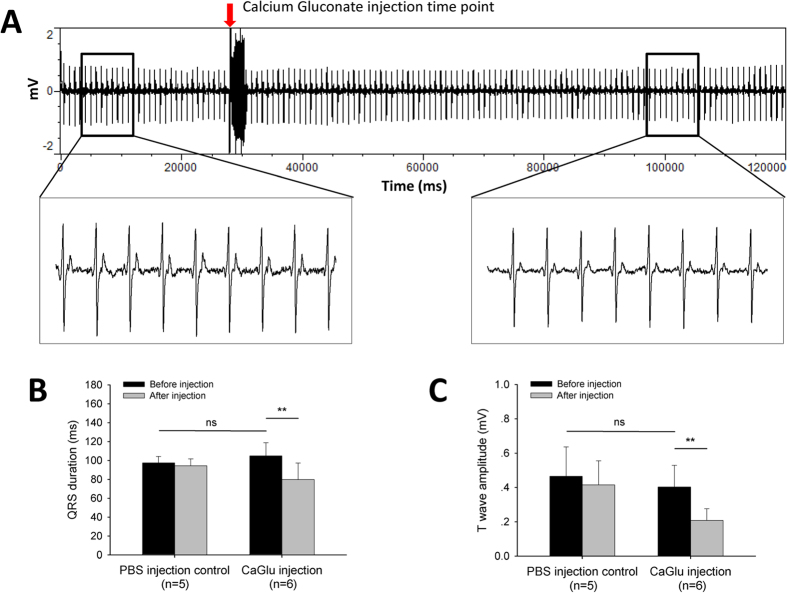
Calcium gluconate rescues hyperkalemia in adult zebrafish. (**A**) Simultaneous recording of ECG during intraperitoneal calcium gluconate injection and the waveform before and after injection. (**B**) QRS duration and (**C**) T wave amplitude before and after injection. Values are presented as the mean + SD, **p < 0.01, pair sample two-tailed t-test.

The baseline ECG signal showed a normal waveform and distinct ECG component before injection (Fig. 3B). After peritoneal injection of KCl solution, the change in ECG signal was observed within 2 minutes (Fig. 3A). Arrhythmia within 4 minutes of KCl injection included a shift of QRS vector (decrease of positive component along with increase in the negative component of the QRS complex), variation in the RR interval, prolongation of QRS duration, widening of the P wave, atrioventricular block (AV block), and prominent T wave. Two types of AV block were observed in zebrafish injected with KCl. The image in Fig. 3C shows the second degree AV block with a fixed ratio of P waves:QRS complexes = 2:1 (4 out of 8 zebrafish). 2 out of 8 zebrafish presented the ECG signal shown in Fig. 3D, the PR interval progressively increases from one complex to the next, followed by a period of asystole (longer than 2 RR intervals and shorter than 3 RR intervals) after 3 or 4 heart beats; the PR interval is the longest immediately before the asystole and the shortest immediately after the asystole; the largest increase in PR interval duration typically occurred between the first and second beats of the cycle. This ECG mimics the second degree Mobitz I AV Block on a human ECG (Wenckebach Phenomenon). At the end of recording, all individuals showed an ECG waveform with widened QRS complex and peaked T wave (Fig. 3E, upper), similar to a human typical hyperkalemia ECG (Fig. 3E, lower). No zebrafish specimens died after recovery from anesthesia. A second degree AV block with 2:1 fixed ratio was observed in all injected zebrafish specimens 24 hours post-injection (Fig. 3F). Prior to calcium gluconate injection, the loss of P wave, decreased R wave amplitude, widened QRS and peaked T wave were evident in zebrafish injected with KCl (Fig. 4A). The QRS interval (Fig. 4B) was significantly shortened and T wave amplitude was significantly decreased (Fig. 4C) at 1 minute after CaGluc injection. No significant change in QRS interval and T wave amplitude was observed in PBS injection control group (Fig. 4B,C).

### ECG of zebrafish heart injury models during regeneration

**Figure 5:**
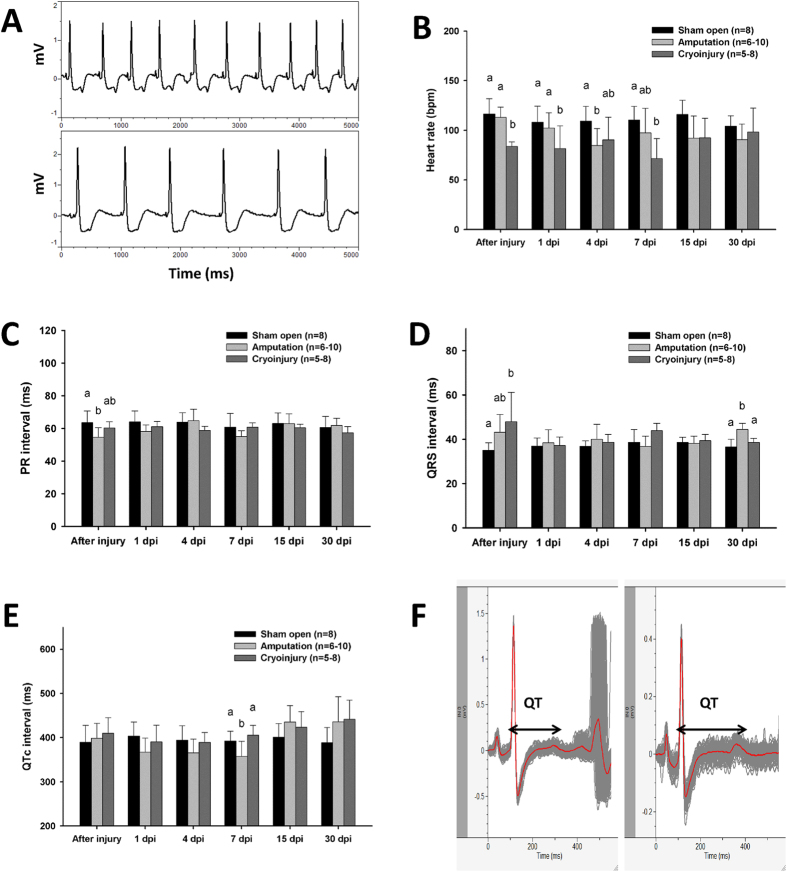
ECG of heart injury models in zebrafish. (**A**) ST segment depression and inverted T wave in amputation (upper) and cryoinjury (lower) model immediately following injury. (**B**) Heart rate, (**C**) PR interval, (**D**) QRS interval, and (**E**) QTc interval of sham control and two injury models during regeneration. Values are presented as the mean + SD, different letters indicate means that are significantly different (Tukey’s test, p < 0.05). (**F**) ECG waveform of 30 days post-cryoinjury zebrafish with prolonged QT interval (right) compared with sham open control (left).

**Figure 6:**
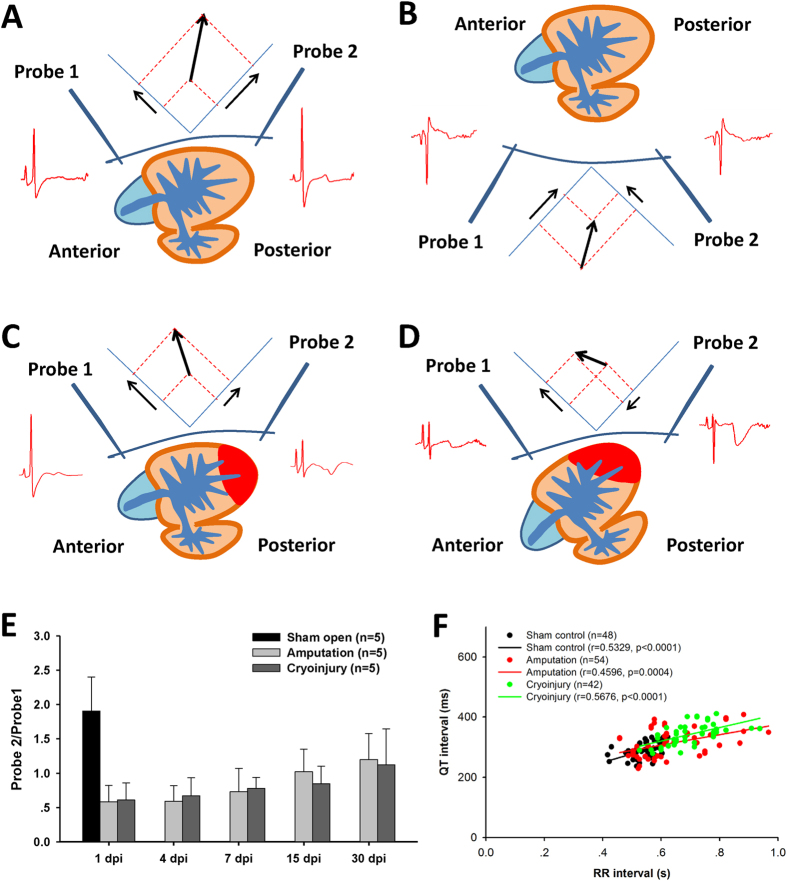
Direction of zebrafish cardiac vector and its shift during heart regeneration. ECG recorded from chest (**A**) and dorsal side (**B**) showing the direction of normal depolarization vector. (**C**) Shift in cardiac vector after ventricular injury. (**D**) Negative-directed R wave occasionally recorded by Probe 2. (**E**) Ratio of two-probe amplitude representing recovery of cardiac vector along with the regenerative process. Values are presented as the mean + SD. (**F**) Correlation coefficient between QT and RR interval of sham control, amputation and cryoinjury group.

ST segment depression and inverted T wave was observed both in amputation and cryoinjury models immediately after injury (Fig. 5A). In contrast to the amputation group that showed a lower heart rate at 4 dpi (days post injury), cryoinjury depressed the heart rate immediately following injury and at 1, 4, and 7 dpi (Fig. 5B). A shortened PR interval was observed in the amputation model after injury (Fig. 5C). Both injury methods induced prolongation of the QRS interval immediately after injury (Fig. 5D). The QRS prolongation was also observed at 30 dpi in amputation model. A slight shortening (not significantly) of the QTc interval in the amputation model was observed at 1 and 4 dpi and significant decrease at 7 dpi. The QTc interval in both injury models was prolonged at 30 dpi compared with the sham operated control although the difference was not significant (one-way ANOVA, *p* > 0.05). QT intervals of the three groups were plotted against the associated RR intervals. Linear regression revealed a significant correlation coefficient between QT and RR interval in all groups (Fig. 6F).

### Shift of cardiac vector in the zebrafish heart regenerative process

The signal amplitude recorded by Probe 2 in wild-type zebrafish was higher than that of Probe 1 (approximately twofold higher, Fig. 6E), indicating that the direction of the zebrafish ventricular vector was toward the posterior, as shown in Fig. 6A. To further observe the vector of zebrafish surface ECG signal, we placed zebrafish face down, and the two probes were placed in the dorsal side above the pericardial cavity. Negative-going ECG signals were then recorded and revealed the amplitude of Probe 1 to be higher than that of Probe 2 (Fig. 6B). During ventricle resection or cryoinjury at the apex (Fig. 6C), the direction of the cardiac vector shifted from the posterior to the anterior (Fig. 6C), leading to greater reduction in amplitude of Probe 2 compared with Probe 1 (Fig. 6E). A negative R wave was occasionally observed in Probe 2 (Fig. 6D). The increase in this ratio was observed in the amputation model within 30 days (*p* = 0.02, Huynh–Feldt test, repeated measure ANOVA) but not in the cryoinjury model (Fig. 6E).

### Cardiac electrophysiology characteristics of breakdance mutant zebrafish

**Figure 7:**
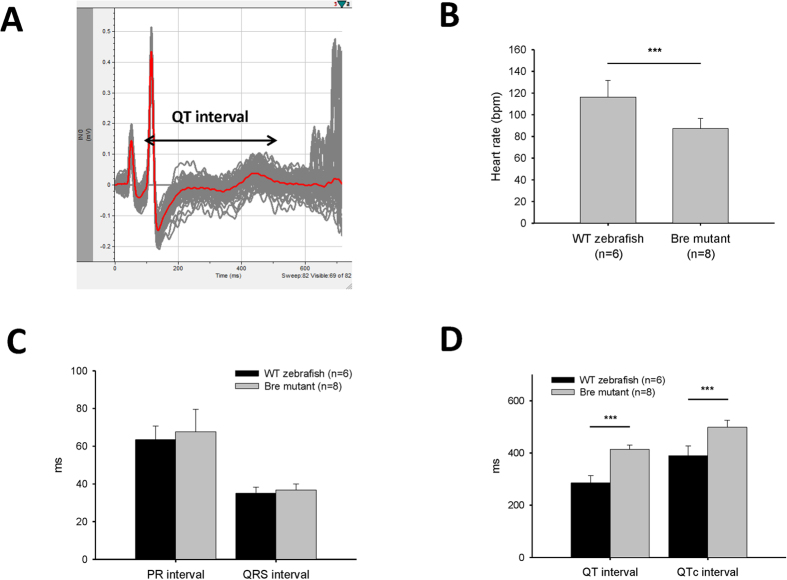
Adult ECG of human long QT syndrome model: breakdance mutant zebrafish. (**A**) ECG Waveform of breakdance mutant with prolonged QT interval. (**B**) Heart rate, (**C**) PR and QRS interval, and (**D**) QT and QTc interval of *bre* mutant compared with wild-type zebrafish. Values are presented as the mean + SD, ***p < 0.001, two-tailed t-test.

The waveform of the breakdance mutant zebrafish ECG signal showed a distinct P wave, QRS complex, and T wave, similar to those of the wild-type zebrafish (Fig. 7A). Nonetheless bradycardia occurred in the *bre* mutant with a heart rate of 87.4 bpm ± 9.3 bpm, significantly slower than that of wild-type zebrafish (Fig. 7B). No significant difference in PR and QRS intervals was detected between *bre* mutant and wild-type zebrafish (Fig. 7C). QT prolongation was observed in *bre* mutant (QT was 414 ms ± 16 ms, QTc was 498 ms ± 27 ms, *n* = 8), and was significantly longer than that of wild-type fish (*p* < 0.001, Fig. 7A,D).

## Discussion

A high quality raw surface ECG signal was obtained in the present study using the modified recording method. A noise-free ECG waveform with distinct P, R, S, and T waves was observed on the average trace of all identified heart beat cycles within a 1 min signal. In contrast to the conventional method of manual measurement on several heart cycles from an ECG recording, we facilitated the accuracy and efficiency of zebrafish ECG analysis by using a common and easily achieved software in electrophysiology study. In our results, the baseline parameters of zebrafish were characterized with a heart rate of 118 bpm ± 14 bpm. The mean PR and QRS interval was 63.5 ± 7.2 and 35.0 ms ± 3.3 ms (*n* = 10), respectively. The QT interval was 282 ms ± 29 ms, and mean QTc was 389 ms ± 38 ms. The QT and QTc values reported in this study are substantially closer to that of a human ECG with the QT interval between 300 and 450 ms. This result agrees with several previous studies but is very different to others (Table 1). In addition to recording and methods of analysis, such variation may be due to the temperature during recording that has been reported as a main factor affecting QT interval in dogs^8^, and in *in vitro* recording of action potentials of zebrafish heart^9^.

In recording a zebrafish ECG, the fish must be immobilized by means of a paralysing agent. Tricaine is a sodium channel blocker that is thought to affect zebrafish heart rate^10^,^11^. Nonetheless it is the only FDA approved anesthetic for market fishes and is widely used by the research community for zebrafish anesthesia. In the present study, we carefully controlled anesthesia depth and operating time to minimize the effect of tricaine. More work is required to determine the cardiac effects of tricaine. In zebrafish ECG recording, the high frequency component of the ECG signal is derived from a 50 Hz power line interference, and the low frequency component represents the muscle and movement artifacts. Therefore, a proper high pass and low pass filter is required to obtain stable and reproducible results. Using a 3 Hz–40 Hz band pass filter and comparing the wandering baseline signal recorded within 1 Hz–100 Hz (Fig. 2H), we obtained a raw ECG signal with an isoelectric baseline, and the 50 Hz power line noise could be further minimized (Fig. 1C). The greatest advantage of our method is the lack of a required signal process. The distinct ECG components, especially T wave, can be easily identified directly from the raw signal. In comparison, the wavelet transform method has been commonly used in previous studies^5^,^7^,^12^. We also demonstrated that the dermis and the silvery pericardial sac affect the surface ECG recording of adult zebrafish. The zebrafish dermis is principally composed of densely packed collagen fibers^13^. Needle electrodes were difficult to insert through this tough layer but the layer can be peeled away easily by forceps. Peeling of the dermis on the chest significantly enhanced signal recording from the muscle layer on the chest. In addition, opening the pericardial sac further enhanced the surface ECG signal without changes to the electrophysiology of the heart. This was evidenced by casper mutant zebrafish in the present study. Casper zebrafish is a mutant line without a reflective iridophore layer^14^, and the silvery pericardial sac is absent. It is possible that the silvery pericardial sac has a similar component and/or structure to the iridophore layer in zebrafish. Our results suggest that this layer affects surface ECG recording in zebrafish, possibly due to the different conductivity of this layer compared with other tissues, although there was no evidence to support this. Based on our observations, pretreatment surgery to open the pericardial sac of zebrafish is highly recommended for ECG recording in the drug screening platform.

Potassium is vital for regulating the normal electrical activity of the heart. Extracellular potassium level in humans is normally maintained in the range of 4.0–4.5 mmol/L. Low (hypokalemia) and high (hyperkalemia) potassium levels lead to abnormal cardiac physiology with potentially life-threatening consequences. Hyperkalemia is defined as a serum potassium level >5.0 mmol/L^15^. Typical ECG findings in hyperkalemia range from peaked T waves, lengthening PR interval, loss of P waves, to widening of the QRS complex, ST segment elevation, ectopic beats and escape rhythm, ventricular fibrillation, and asystole^16^,^17^. In the present study, the zebrafish ECG from hyperkalemia showed surprising similarity to human hyperkalemia ECG. The effect of peritoneal injection of potassium on cardiac physiology was promptly evidenced by ECG changes within 1 minute (Fig. 3). The widening QRS complex indicated an intraventricular conduction delay in the zebrafish heart after KCL injection (Fig. 3). Second degree AV block with a fixed ratio and Mobitz I rhythm were observed in the treated zebrafish (Fig. 3C,D). Instead of a non-conductive P wave at the end of each heart beat group in the human Mobitz I rhythm, asystole, which is a life-threatening rhythm in severe human hyperkalemia, occurs in zebrafish. An increase in the serum potassium level in humans also leads to a reduction in the amplitude of the P wave^18^. This response similarly occurs in zebrafish, especially 24 hours following injection of KCl and in the presence of second degree AV block (Fig. 3F). Calcium gluconate is commonly used as the first-line treatment of human hyperkalemia^19^. The intravenous calcium takes effect within 1 to 3 minutes and lasts for only 30 to 60 minutes in humans^20^, and was highly comparable in this study with zebrafish peritoneal injection. Additionally, the changes in QRS duration and T wave amplitude indicated that calcium may rescue hyperkalemia in the zebrafish by a similar mechanism to that in humans, such as membrane stabilization. Hemodialysis and ion exchange resins are used in the clinical treatment of human hyperkalemia to remove potassium from the body^20^. In the zebrafish, the QRS and T wave recovered to normal within hours (data not shown), possibly due to the high efficiency of potassium excretion through the gills^21^. To the best of our knowledge, we are the first to reveal the potential of zebrafish as a model of human hyperkalemia. Hyperkalemia occurs as a side effect of certain medicines due to a variety of mechanisms^22^. Our results provide valuable insight into the application of zebrafish in drug safety screening and as a means to study drug-induced hyperkalemia.

In the present study, we report the novel observation of ST segment depression in the zebrafish ECG immediately after injury. In numerous instances, ST segment depression is associated with acute ischemia and acute infarction^23^. The resection of zebrafish ventricular apex in the amputation model lead to hemorrhage that may mimic acute cardiac ischemia. On the contrary, cryoinjury mimics acute infarction in humans. Both amputation and cryoinjury prolongation of QRS immediately after injury indicated an acute disturbance to the intraventricular conduction systems during heart injury. The shortened QTc interval has been reported at 4 days post-amputation in a previous study^7^, and prolonged QTc has been reported in longitudinal observation of the same injury model for approximately two months^12^. In our results, the QTc interval significantly shortened at 7 days post amputation (dpa), and was prolonged at 15 and 30 dpa, albeit not to a significant degree. This may have been due to the massive cardiomyocyte proliferation that occurred from days 7 to 14 dpa in the amputation model^24^. That such change was not observed in the cryoinjury model implies a slower regeneration process and is consistent with the results of histological studies^25^,^26^. In the cryoinjury model, a modest trend in QTc prolongation was observed at 30 days post cryoinjury (dpc), whereas QTc prolongation has been reported at 7 dpc but not at 30 dpc in previous study^27^. The debate about cardiac electrophysiology during heart regeneration highlights the necessity for a reliable and reproducible standardised method for zebrafish ECG recording and analysis.

Unlike the compact structure of a four-chamber human heart, the structure of the zebrafish heart is tubular, with the atrium and ventricle suspended in the fluid inside the pericardial cavity. This difference may explain the difficulty in achieving successful and reproducible ECG recordings in zebrafish^3^. The positions of the two probes in the present study mimicked the V4 and V5 (or V6) precordial leads in a human ECG, and allowed us to detect the mean vector of ventricle excitation that is pointed toward the chest surface and the posterior (Fig. 2A). Moreover, the atrium vector (P wave) is in the same direction as the ventricle. This finding disagrees with those of previous studies, in which opposite amplitudes of P and R waves were reported in *in vivo* and *in vitro* recording models^6^,^28^,^29^. The negative ECG signals of P and R waves in the dorsal probe (Fig. 2B) also demonstrated that the projection of ventricular vector on the two axes is relatively stable. When the ventricular apex was resected or cryoinjured, the vector of ventricle excitation single shifted to the anterior, thereby rendering the amplitude of Probe 1 higher than that of Probe 2 (Fig. 6C). A negative R wave vector with a positive P wave vector was observed in Probe 2, occasionally in both injury models (different to the negative-going signal from the dorsal recording probes). These sporadically detected vectors may have been due to variation in the injury site and orientation of the ventricle inside the pericardial cavity. Notably, recovery of the ventricular vector occurred within one month in the amputation model, while the cryoinjury model required a longer time to recover. These results are also in strong agreement with evidence from the histological study of these injury models that reveal that the regeneration process in the amputation model is faster than that of the cryoinjury model^30^,^31^. It also reflected the sensitivity of the improved zebrafish ECG recording method. Furthermore, the significant correlation coefficient between QT and RR interval demonstrated the reproducibility of the recording and analysis method.

The QT interval in humans is 300 to 450 ms. This interval can be prolonged in the presence of an ion channel disorder and may lead to ventricular tachyarrhythmia and sudden death^32^,^33^. Mutations in human HERG (ether-à-go-go-related gene), that encodes the rapidly activating delayed rectifier K^+^ channel, are associated with human chromosome 7-linked congenital long QT (LQT-2) syndrome^33^. Zebrafish breakdance is one of the most reported mutants on *erg* and presents an AV block and LQST in the embryo stage^1^,^29^,^34^. Although the ECG of adult *bre* mutant has been previously reported^29^, the waveform shows a non-typical ECG signal even in wild-type zebrafish (Table 1, ref. 29). Nonetheless LQTS has still been reported for homozygous *bre* mutant with the QTc of wild-type and homozygous *bre* mutant 427 ± 35.7 and 572 ms ± 23.8 ms, respectively. Both values are longer than those in the present study (414 ± 16 and 498 ms ± 27 ms). In addition, bradycardia occurs in zebrafish *bre* mutant with a heart rate of 87.4 bpm ± 9.3 bpm and indicates the lower cardiomyocyte excitability due to ion channel disorder. Instead of 2:1 AV block in the embryo *bre* mutant, the mutation on the potassium ion channel does not affect atrial ventricular conduction in adults. Our work describes the cardiophysiology of the *bre* mutant using a modified method that explored the potential of this zebrafish mutant in the study of human congenital LQTS and the function of ion channels in heart regeneration.

Although zebrafish is emerging as an animal model for *in vivo* pharmacological assessment and cardiovascular study, most of studies have used embryo not adult zebrafish. The lack of adult zebrafish ECG study is mainly due to the absence of a reliable recording method for quantifying cardiac parameters. Researchers have doubted the suitability of the zebrafish ECG in its current form as a means to investigate or simulate other cardiac disorders^3^. The proposed multi-electrode method in the present study is expected to contribute significantly to the field of zebrafish heart regeneration and *in vivo* screening of cardiovascular drugs. In summary, we improved the surface ECG recording and methods of analysis, and provided evidence of its sensitivity, reliability and reproducibility. The potential of adult zebrafish and its ion channel disorder mutant as a model of the human cardiovascular system exceeded our expectations.

## Methods

### Zebrafish husbandry and management

Wild-type zebrafish (*D. rerio*) were procured from a local aquarium. All zebrafish including AB line, casper and breakdance mutant were maintained in a controlled environment at the City University facility, at a temperature of 28 °C and a light/dark cycle of ~14 h/10 h. Adult (10 to 12 months old) zebrafish were selected for the study. All animal protocols in this study were approved by Department of Health, Hong Kong SAR, China (refs (9, 10, 11, 12, 13, 14, 15) in DH/HA&P/8/2/5 Pt.3). All experiments were performed in accordance with relevant guidelines and regulations in Hong Kong SAR, China.

### Surface ECG recording system

An ECG was recorded in adult zebrafish using a protocol modified from Milan *et al.*^2^. Fish were anesthetized carefully by immersion in 0.02% tricaine solution. Once gill movement stopped, the fish was placed on a damp sponge with the ventral surface uppermost. Scales covering the chest area were carefully removed. Two stainless steel electrodes were inserted into the muscle layer of the chest to a depth of approximately 0.5 mm. Probe 1 was placed in an anterior position, while Probe 2 was located on the posterior. The positions of both electrodes are shown in Fig. 1B. The reference electrode was positioned along the midline posterior to the pelvic fin. ECG signals were amplified by a differential amplifier (Axon Multiclamp 700B, Molecular Devices) with 1000 gain value and digitalized by Digidata 1440A (Molecular Devices) at a sampling rate of 2,000 Hz in gap-free mode (Fig. 1A). A 3 Hz–40 Hz band pass filter was used to remove muscle movement and power line artifacts. Whole processing time, which included recording time, was controlled to within 5 minutes after which the fish was returned to water to recover from anesthesia. All experiments were conducted at room temperature (25 °C). Off-line data processing was performed with Clampfit 10.0 (Molecular Devices).

### ECG signal analysis and intervals measurement

Analysis of the raw ECG signal was conducted semi-automatically using the template search function of Clampfit 10.0 software (Molecular Devices) (Fig. 1D). A complete heart beat cycle presenting distinct P, R and T wave was identified manually and selected as a template (500 to 800 ms signal). This template was then used as a reference waveform in template search analysis. The signals identical to the template were extracted, overlaid and aligned to the highest amplitude (R wave) automatically (gray trace in Fig. 1D). An average trace was generated using the software (red trace in Fig. 1E). The measurement of all ECG intervals, including PR, QRS, and QT, was conducted manually on the average trace (Fig. 1E). PR interval is defined as the time from the upstroke of P wave to the upstroke of the R wave (Fig. 1E). Since the Q wave is absent and J point is un-identifiable in zebrafish ECG, QRS interval was defined in this study as the time from the upstroke of the R wave (the onset of depolarization) to the negative peak of the S wave (Fig. 1E). QT interval is defined as the time from the upstroke of the R wave to the end of the T wave (termination of repolarization) (Fig. 1E). The mean QT interval was calculated when the T wave was identifiable in both two probes. Otherwise, the value from one probe was recorded. RR interval is defined as the time between the peaks of two consecutive QRS complexes. The interval was measured automatically by the threshold search function of the Clampfit 10.0 software (Molecular Devices). QT intervals were then normalized to heart rate using the standard Bazett formula: QTc = QT/(RR^1/2^).

### Effects of dermis and pericardial sac on ECG measurement

Four groups of wild-type zebrafish were prepared to investigate the effects of dermis and pericardial sac on surface ECG recording. Scales covering the chest area were removed, and the two electrodes were placed on the chest (group 1). In group 2, after scale removal, the electrodes were inserted through the dermis to a depth of approximately 0.5 mm. In group 3, after scale removal, the dermis on the chest between the two electrodes was peeled off, and electrodes were inserted into the muscle layer. In group 4, zebrafish pretreatment was conducted one week before ECG was recorded. An opening of about 0.25 mm^2^ was carefully torn in the pericardial sac using forceps (Fig. 1B). After one week, the wound was healed, whereas the pericardial sac did not heal. Before ECG was recorded, the dermis was also peeled, and the electrodes were inserted into the muscle layer for recording. In addition, the casper mutant zebrafish without silvery pericardial sac was used to further validate our results. In group 5, the electrodes were inserted through the dermis. In group 6, the dermis on the chest between two electrodes was peeled off. The waveform and amplitude of R wave from Probe 2 in raw ECG signals were compared between each group.

### Hyperkalemia ECG of adult zebrafish and the rescue effects of calcium gluconate

To further demonstrate the potential of zebrafish as a human cardiovascular model, we investigated the effects of hyperkalemia on zebrafish cardio electrophysiology and the similarity between zebrafish and human hyperkalemia ECG. The pericardial sac of wild-type zebrafish (n = 8) was opened one week before KCl injection. Zebrafish were anesthetized carefully by immersion in 0.02% tricaine solution and placed with the ventral surface uppermost on a damp sponge. ECG signal was recorded as described above. Baseline ECG signal was recorded for 1 min. Then, 10 μL of 200 mM KCl was injected into the peritoneal cavity, and ECG signal was recorded simultaneously for 5 min. Fish were then placed in water to recover from anesthesia. To investigate the rescue effect of CaGluc (Calcium gluconate) on the arrhythmia caused by hyperkalemia, zebrafish were injected with 10 μL of 200 mM KCl first to induce hyperkalemia. Once hyperkalemia ECG (widened QRS, peaked T wave, Fig. 3E upper) was observed, the signal was recorded for 30 seconds before injecting the zebrafish with 10 μL of 100 mM CaGluc peritoneally, and the ECG signal was recorded continually for a further 4 minutes. 10 μL of PBS was injected in the control group.

### Zebrafish ECG during heart regeneration in two injury models

AB line zebrafish specimens were anesthetized carefully by immersion in 0.02% tricaine solution and placed with the ventral surface uppermost on a damp sponge. The chest was opened at the location shown in Fig. 1B. Heart amputation and cryoinjury were conducted in treatment groups (10 to 12 fish specimens for each group) using the method described in previous studies^24^,^38^. Sham-operated fish specimens without heart injury were prepared as the control group (8 fishes). After injury and sham operation, the ECG signal was recorded for 1 min immediately as described above. Fish specimens were then placed in water to recover from anesthesia. At 1, 4, 7, 15, and 30 days post-injury (dpi), ECG signal of injured zebrafish by both injury methods and sham control zebrafish was recorded. Heart rate, PR, QRS, and QT intervals were measured as described above.

### ECG Characteristics of breakdance zebrafish mutant

The adult homozygous *breakdance* (*bre*) mutant fish were screened by genotyping. The *bre* mutant zebrafish specimens were then anesthetized carefully by immersion in 0.02% tricaine solution and placed with the ventral surface uppermost on a damp sponge. The chest of zebrafish was opened at the location shown in Fig. 1B, and the pericardial sac was opened as described above. The ECG signals of zebrafish mutants were recorded after one week as described above. Heart rate, PR, QRS, and QT intervals were also measured.

### Statistical analysis

Normality and homogeneity of variance of the data were initially checked to confirm parametric assumptions. Effects of pretreatment method and probe insertion on R wave amplitude were tested by one-way ANOVA followed by Tukey’s test of multiple comparisons. Considering that the zebrafish samples were repeatedly observed in the experiment, repeated measure ANOVA was used to detect the recovery of cardiac vector in injury models. Rescue effect of CaGluc was tested by paired sample t-tests. Differences between wildtype and breakdance mutant ECG were tested by independent sample t-tests. All statistical tests were performed using the statistical software, SPSS 13.0 with the significance value set at p = 0.05.

## Additional Information

**How to cite this article**: Liu, C. C. *et al.* Improvement of surface ECG recording in adult zebrafish reveals that the value of this model exceeds our expectation. *Sci. Rep.* **6**, 25073; doi: 10.1038/srep25073 (2016).

## Figures and Tables

**Table 1:**
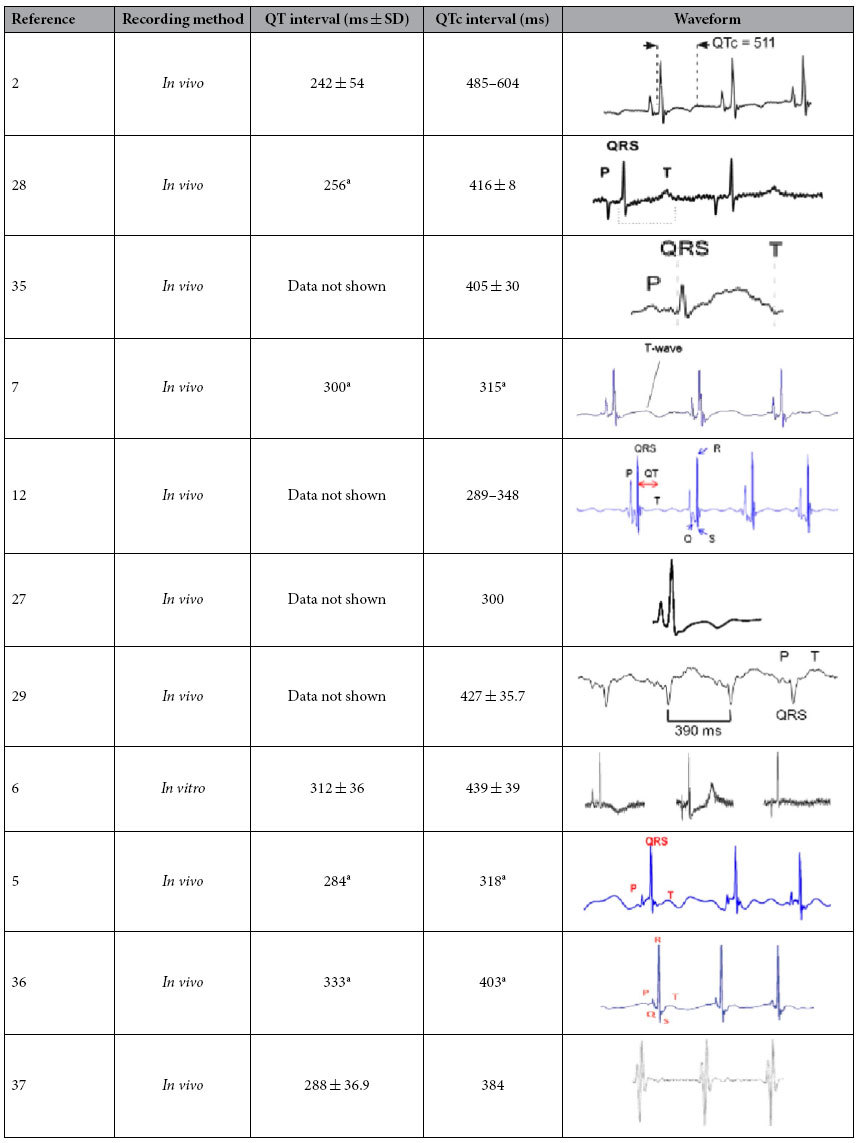
Zebrafish ECG waveform, QT, and QTc interval in previous studies.

^a^RR and QT interval were measured from the waveform with time axis in the paper; QTc was calculated by Bazett’s formula.
